# Efficacy and safety assessment of propranolol tablets vs. oral solution for infantile hemangioma: a retrospective study in China

**DOI:** 10.3389/fped.2025.1542348

**Published:** 2025-02-04

**Authors:** Wenting Chen, Hua Qian, Qi Sun, Shan Zhang, Lei Zhu, Yafen Wu, Yingying Qian, Bingbing Wang, Wei Li

**Affiliations:** Department of Dermatology, Children's Hospital of Soochow University, Suzhou, China

**Keywords:** propranolol hydrochloride oral solution, propranolol hydrochloride tablets, infantile hemangioma, safety, efficacy

## Abstract

**Background:**

Propranolol for infantile hemangiomas (IHs) is effective and relatively safe. However, propranolol has different formulations and there is no consensus on the optimal formulation for IHs. The propranolol oral solution was not used in China until 2022.

**Objective:**

To evaluate the efficacy and safety of propranolol tablets and an oral solution in infants with high-risk IH.

**Methods:**

A retrospective cohort study was conducted involving 234 consecutive patients with a clinical diagnosis of high-risk IH who were treated with propranolol between August 2018 and February 2023 (propranolol tablets, 168 patients; propranolol oral solution, 66 patients). All patients were assessed in the hospital at the initiation of treatment and in the outpatient setting during treatment. The Hemangioma Activity and Severity Index was used to monitor the clinical activity of the hemangioma after propranolol treatment.

**Results:**

Based on the Hemangioma Activity and Severity Index, 66.52% and 69.15% improvement occurred in the propranolol tablet and oral solution groups, respectively. 23.21% of patients in the propranolol tablet group and 42.42% in the oral solution group achieved >75% score improvement (*X*^2^ = 8.557; *P* = 0.003). Adverse reactions occurred in 34 (20.24%) and 11 patients (16.67%) in the propranolol tablet and oral solution groups, respectively. The most common adverse reaction in the propranolol tablet group was liver function abnormalities due to mild elevation of liver enzymes (*X*^2^ = 4.09; *P* = 0.045).

**Conclusion:**

Both propranolol tablets and oral solution had positive efficacy in patients with high-risk IHs, but more patients in the propranolol oral solution group achieve >75% score improvement compared to the propranolol tablet group. No life-threatening adverse reactions occurred in either group but liver function abnormalities were more likely to occur in patients treated with propranolol tablets.

## Introduction

Infantile hemangiomas (IHs) are vascular tumors characterized by endothelial-like cell proliferation. IHs are the most common benign tumor in children, are estimated to occur in 4%–5% of children (1), and occur more frequently in preterm infants with low-birth-weight, females, and Caucasian populations (2). According to the natural history of lesions, IHs can be divided into proliferative, stable, and receding phases. Although most IHs spontaneously regress, some IHs are associated with complications that can be life-threatening, impair function, and require aggressive treatment. In 2008 Léauté-Labrèze (3) first reported that the β-blocker, propranolol, is beneficial in the treatment of IHs. Since then numerous randomized clinical trial studies have documented the efficacy and safety of propranolol for IHs (4). Moreover, propranolol has replaced glucocorticoids as first-line therapy for high-risk IHs (5, 6). In 2014, the original propranolol oral solution [propranolol hydrochloride (Hemangeol), 3.75 mg/ml oral solution; Pierre Fabre Dermatologie, Boulogne-Billancourt, France] became the first drug with clear indications for the treatment of IHs. Until 2021 the propranolol oral solution, Hemeijia (propranolol hydrochloride, 120 ml: 450 mg oral solution; Wuhan Kefu New Pharmaceutical, Wuhan, China), was approved for marketing by the State Drug Administration (SDA). Since that time the oral solution dosage form of propranolol has been gradually utilized to treat IHs in China. However, the efficacy and safety of traditional propranolol hydrochloride tablets and new propranolol hydrochloride oral solutions for IHs have not been compared. Therefore, a retrospective study was conducted to characterize the efficacy and safety of propranolol tablets and solutions to treat IHs in a large cohort.

## Materials and methods

### Clinical information

A retrospective cohort study was conducted in the Department of Dermatology (Children's Hospital of Soochow University, Soochow, China) involving 234 patients with a clinical diagnosis of high-risk IHs requiring propranolol treatment (7) between August 2018 and February 2023. One hundred sixty-eight patients with 193 hemangiomas were treated with propranolol hydrochloride tablets (10 mg/tablet; Jiangsu Yabang Aiperson, Jiangsu, China) and 66 patients with 84 hemangiomas were treated with propranolol oral solution (Hemeijia, 120 ml: 450 mg oral solution; Wuhan Kefu New Pharmaceutical, Wuhan, China). Three patients switched from propranolol hydrochloride tablets to the oral solution and were excluded from the study. High-risk IH patients in whom there were contraindications to treatment with a β-blocker, such as hypotension, bradycardia, hypoglycemia, II–III degree heart block, cardiogenic shock, severe left ventricular cardiac insufficiency, bronchospasm, and allergic rhinitis (8) were not eligible to participate in the study. The study was approved by the hospital Ethics Board (2024CS077) and families provided written informed consent.

### Treatment

Propranolol tablets were administered every 8 h orally, as follows: 0.5 mg/(kg·day) on the 1st day; 1.0 mg/(kg·day) on the 2nd day; and gradually increased to the maintenance target dose of 1.5 mg/(kg·day) on the 3rd day. All patients were administered propranolol in the outpatient observation room daily until on a maintenance dose. The propranolol oral solution was administered every 12 h, as follows: 0.5 mg/(kg·day) initial dose for preterm infants; 1.0 mg/(kg·day) for full-term infants; and a 2-week follow-up evaluation after exclusion of contraindications was performed at a maintenance dose of 1.5 mg/(kg·day) for preterm infants and 2.0 mg/(kg·day) for term infants, which was compliant with the international guideline (9, 10). All patients were observed in the outpatient room when taking the initial dose.

The method of taking propranolol tablets was as follows: 10 mg propranolol tablets were thoroughly dissolved in 10 ml of water (1 ml of dissolved liquid is equivalent to 1 mg of propranolol) and placed in a 10-ml syringe; the syringes were stored in a refrigerator at 4℃; and propranolol was administered according to the required dosage. Hemeijia, a propranolol oral solution, was supplied with an oral dosing syringe for administration. Hemeijia was administered directly into the child's mouth. If necessary, Hemeijia was diluted in a small quantity of milk or fruit juice and administered in baby bottles.

The heart rate, blood pressure, and blood glucose level were monitored in the outpatient clinic after administration of propranolol tablets or propranolol oral solution on days 1–3. Self-monitoring was performed during the administration of propranolol at home. The parents returned to the clinic 14 days after the start of treatment and the following were reviewed: electrocardiogram; ultrasound findings; blood counts; blood coagulation profile; cardiac enzyme profile; thyroid, liver, and renal function; and blood glucose level. The follow-up interval was monthly with an electrocardiogram and ultrasound performed at each visit.

The treatment endpoints were as follows: (1) tumor regression >75%; (2) no tumor growth during three consecutive monthly follow-up evaluations; and (3) ultrasound findings that color Doppler flow imaging disappeared or was not apparent. Achieving one or more endpoints was consistent with satisfactory treatment.

### Assessment of efficacy

The Hemangioma Activity and Severity Index (HASI), which was developed by Semkova in 2015 (11), was used to evaluate IH preliminary validation after propranolol. Improvement in IH was quantified as the percentage difference in scores between the beginning and end of treatment. Additionally, efficacy was evaluated based on the ultrasound finding of IH depth difference between the beginning and end of treatment.

### Assessment of safety

Propranolol safety was serial assessment (14 days after the 1st dose and during the monthly follow-up evaluations for every month): electrocardiogram; ultrasound images; routine blood tests; blood coagulation profile; myocardial enzyme profile; thyroid, liver, and kidney function; and blood glucose level, which was advised by authoritative international guideline (12). Patients were advised to have a sphygmomanometer, blood glucose meter, thermometer, and stethoscope available to monitor changes in heart rate, respiration rate, blood pressure, and blood glucose level before and 1 h after propranolol administration.

### Statistical analysis

The variables are described as averages and minimum and maximum values. The statistical differences in IH score improvement and adverse reaction incidence between the tablet and solution groups were performed using a chi-square test. Comparisons between the HASI scores in the two groups were performed using independent and paired samples *t*-tests. Statistical analysis was carried out using SPSS v.25.0 statistical software (IBM Corporation, Armonk, NY, USA). Data visualization was carried out using OriginPro2019 and R studio software. *P* values were two-tailed with a significance threshold of .05.

## Results

### Patients, lesions, and treatment characteristics

Our study included 234 patients [165 females (70.5%) and 69 males (29.5%)]. One hundred sixty-eight of 234 patients (71.8%) received propranolol hydrochloride tablets and 66 of 234 patients (28.2%) received a propranolol hydrochloride oral solution. In both the tablet and oral solution groups, female patients outnumbered male patients.

The total number of evaluated IHs was 277, including 193 in the tablet group and 84 in the oral solution group. Most lesions were located on the face and trunk. Most of the facial hemangiomas were periocular and perioral. It is common to treat parotid IHs with propranolol. The characteristics of patients are shown in Table 1.

**Table 1:** Basic characteristics of the 234 IH patients.

	Propranolol tablet group *n* = 168 (193)	Propranolol solution group *n* = 66 (84)
Gender, *n* (%)		
Male	51 (30.4)	18 (27.3)
Female	117 (69.6)	48 (72.7)
Age at treatment initiation(months)		
Max	17.9	9.5
Min	1.1	1.2
Average	4.1	3.4
Treatment duration(months)		
Max	28.3	18.4
Min	2.33	1.8
Average	11.6	9.4
Treatment duration, *n* (%)		
≤6 months	17 (10.1)	11 (16.7)
6–12 months	82 (48.8)	42 (63.6)
>12 months	69 (41.1)	13 (19.7)
Localization, *n* (%)		
Facial	71 (36.8)	34 (40.5)
*Periocular*	*19* (*9.8)*	*5* (*6.0)*
*Perinatal*	*14* (*7.3)*	*6* (*7.1)*
*Perioral*	*17* (*8.8)*	*11* (*13.1)*
*Periotic*	*5* (*2.6)*	*3* (*3.6)*
*Parotid*	*10* (*5.2)*	*4* (*4.8)*
*Other*	*6* (*3.1)*	*5* (*5.9)*
Scalp or neck	18 (9.3)	11 (13.1)
*Scalp*	*8* (*4.1)*	*7* (*8.3)*
*Neck*	*10* (*5.2)*	*4* (*4.8)*
Trunk	72 (37.3)	27 (32.1)
*Breast*	*7* (*3.6)*	*3* (*3.6)*
*Other*	*65* (*33.7)*	*24* (*28.5)*
Extremities	29 (15.0)	12 (14.3)
Genitalia	3 (1.6)	0 (0.00)

Values are presented as a number (percentage).

Facial consists of Periocular, Perinatal, Perioral, Periotic, Parotid and other, which were highlighted in Italic values. Scalp or neck was separated into Scalp and Neck for statistical description, which were highlighted in Italic values. Because IHs in the breast comprised a large percentage, IHs in Trunk were separated into Breast and Others for statistical description, which was also highlighted in Italic values.

The treatment duration was between 2.33 and 28.3 months (median, 11.6 months) for the propranolol tablet group and between 1.8 and 18.4 months (median, 9.4 months) for the oral solution group. Treatment of patients with propranolol oral solution was generally shorter in duration compared to treatment with propranolol tablets. Most of the children were treated for 6–12 months in the propranolol hydrochloride tablet (48.8%) and oral solution groups (63.6%). All children completed the course of regular medication and did not receive any other treatment during the study. All data are shown in Table 1.

### Efficacy assessment

Based on a comparison of photographs of hemangioma tumors in the two groups before and after treatment, efficacy assessment was evaluated using the HASI score developed by Semkova. The initial score attributed to IH activity at the beginning of treatment was designated as t_0_ and the score at the end of the therapy was designated as t_f_. The difference between the initial and final scores was estimated by the following formula to assess efficacy: (t_0_–t_f_) t_0_.

The severity score difference in the propranolol tablet group and oral solution groups was not significant (*t* = 0.075; *P* = 0.943). The initial activity score for the tablet group at the beginning of treatment (t_0_) ranged from 3 to 15.5 (median, 9.94), while the score at the end of the therapy (t_f_) ranged between 0 and 12 (median, 3.40). The t_0_ ranged from 4 to 14.5 in the oral solution group (median, 10.24), while the t_f_ ranged between 0 and 12 (median, 3.35).

Most IHs improved with propranolol treatment independent of the formulation. Specifically, only 8.33% and 6.06% of patients in the tablet and oral solution groups had a score improvement <25%, respectively. Of the 168 patients in the tablet group, 31 had complete remission (100%). Of 66 patients in the oral solution group, 9 had complete remission (100%).

**Figure 1:**
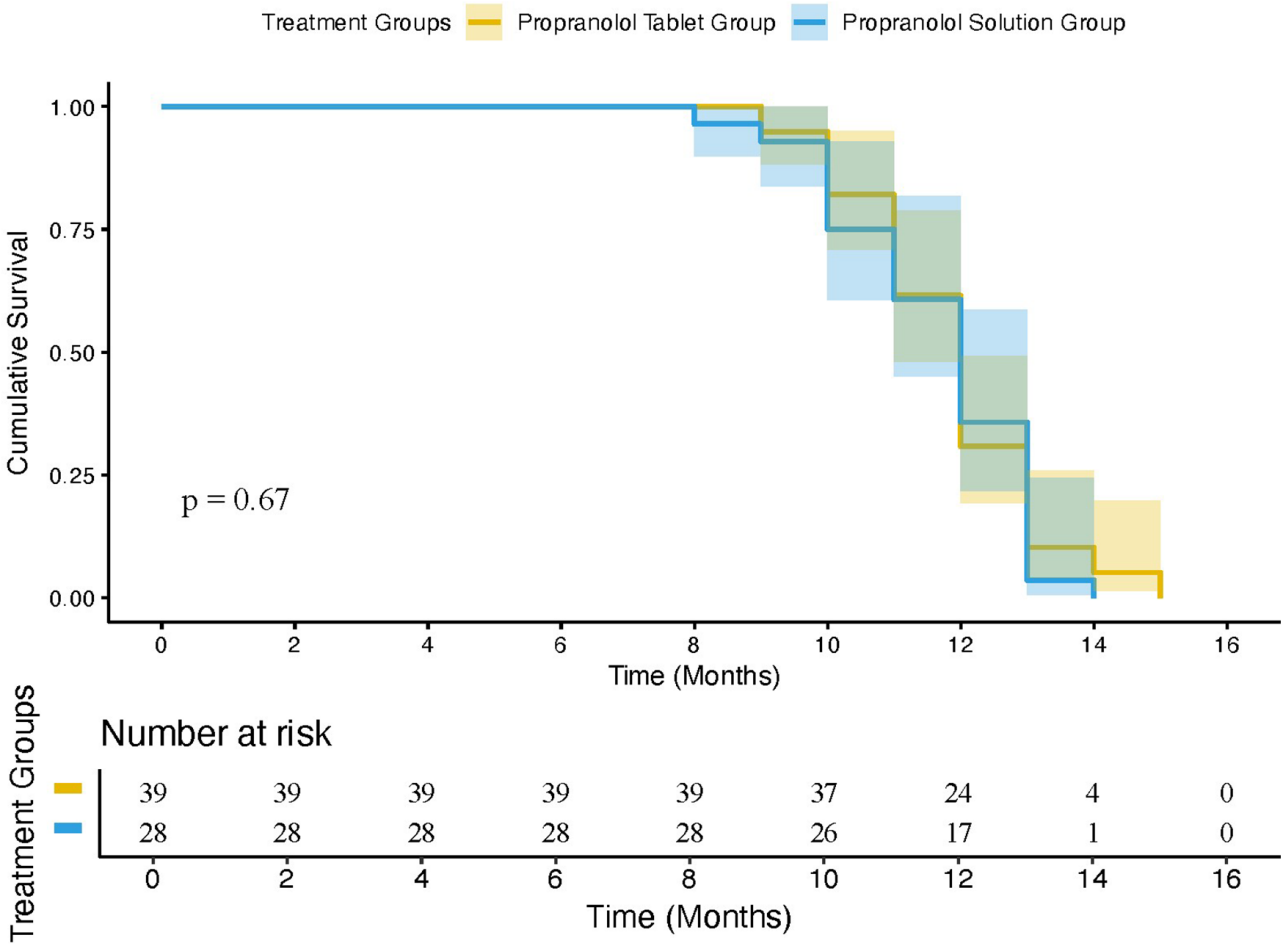
Kaplan-Meier curves for treatment duration to 75% improvement.

The median final scores in the tablet and oral solution groups were 3.40 and 3.35, respectively, with no statistical significance between the 2 groups (*t* = 0.853; *P* = 0.898). The median improvement, expressed as a percentage, was 66.52% and 69.15%, respectively, suggesting no statistically significant difference in therapeutic effect between propranolol tablets and oral solution (*t* = 0.746; *P* = 0.456). As Table 2 shows, most patients (35.71%) who were treated with propranolol tablets achieved a score improvement of 50%–75%, and most patients who were treated with the oral solution (42.42%) achieved a score improvement of 75%–100%. Only 23.21% of patients in the tablet group achieved a score improvement of 75%–100% (*X*^2^ = 8.557; *P* = 0.003). All data are shown in Table 2, and Kaplan-Meier Analysis of the Proportion of Patients who achieved 75% improvement is shown in Figure 1, while the log-rank *P* = 0.67, HR = 1.15(95% CI:0.7–1.88), lacks statistical significance, which means no significant difference in the duration of treatment to achieve 75% improvement in either the tablet or solution groups.

**Table 2:** Efficacy assessment based on the HASI score.

	Propranolol tablet group *n* = 168	Propranolol solution group *n* = 66	*p*-value	*OR (95% CI)*
HASI (severity)				
Score*	2.60	2.59	0.94	
HASI (activity)				
Score t_0_*	9.94	10.24	0.40	
Score t_f_*	3.40	3.35	0.90	
Score t_f_–t_0_*	−6.54	−6.89	0.28	
Score improvement	66.52%	69.15%	0.46	
100%**	*31* (*18.45)*	*9* (*13.64)*	*0*.*38*	0.698 (0.312–1.559)
75%–100%**	*39* (*23.21)*	*28* (*42.42)*	*0*.*00*	*2.437* (*1.330*–*4.465)*
50%–75%**	*60* (*35.71)*	*18* (*27.27)*	*0*.*22*	*0.675* (*0.361*–*1.263)*
25%–50%**	*25* (*14.88)*	*7* (*10.61)*	*0*.*39*	*0.679* (*0.278*–*1.655)*
≤25%**	*13* (*7.74)*	*4* (*6.06)*	*0*.*66*	*0.769* (*0.241*–*2.451)*

* Values are presented a mean. **Values are presented as a number (percentage) and using t-tests.

** Values were highlighted in Italic, We categorized Score improvement into these five grades.

### Safety assessment

The incidence of adverse reactions was 20.24% (34/168 patients) and 16.67% (11/66 patients) for the propranolol tablet and oral solution groups, respectively. Patients treated with propranolol tablets were more likely to have adverse reactions than patients treated with the propranolol oral solution, although not statistically significant.

The most common adverse reaction in the tablet group was liver function abnormalities (15/168 patients), including slight elevations in the alanine aminotransferase or aspartate aminotransferase levels. A minor liver function abnormality occurred in 1 of 66 patients (1.52%) in the oral solution group (*P* = 0.045). Most of the patients in the oral solution group (15/16) were treated with compound glycyrrhizin tablets for several days and one patient recovered spontaneously after 2 weeks. All drug-induced adverse reactions were mild and did not interrupt propranolol treatment.

**Figure 2:**
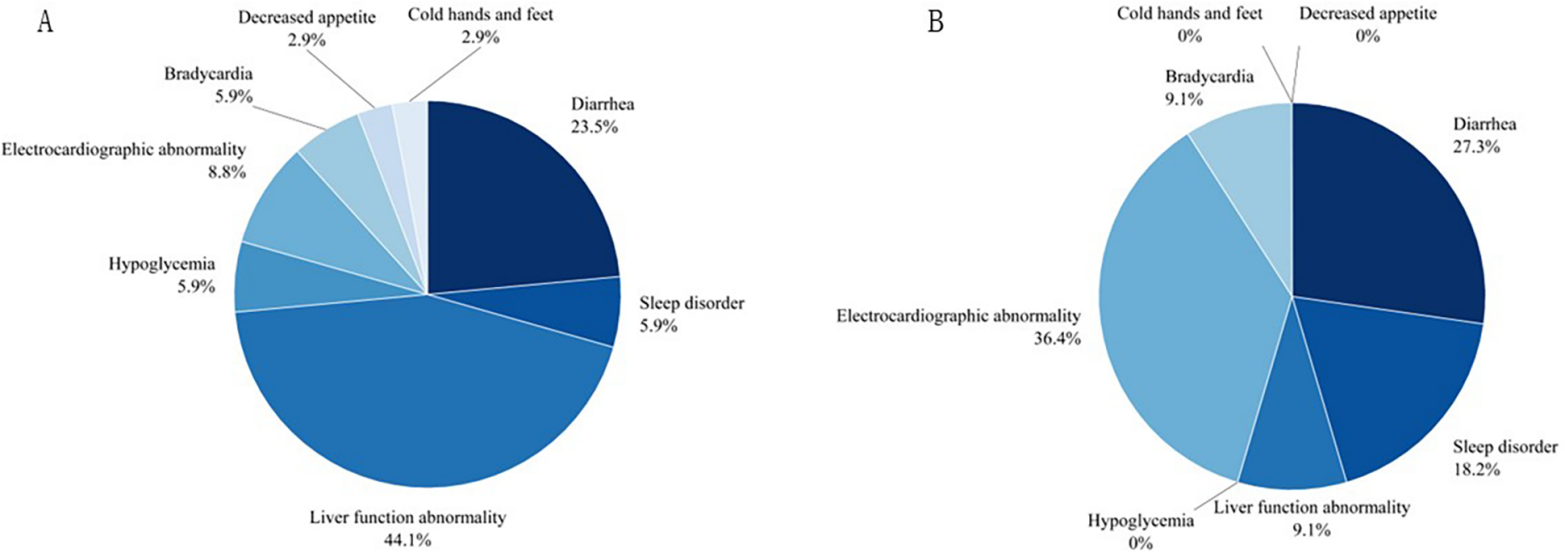
The pie chart on adverse events for two groups. **(A)** For propranolol tablet group, **(B)** for propranolol solution group.

**Figure 3:**
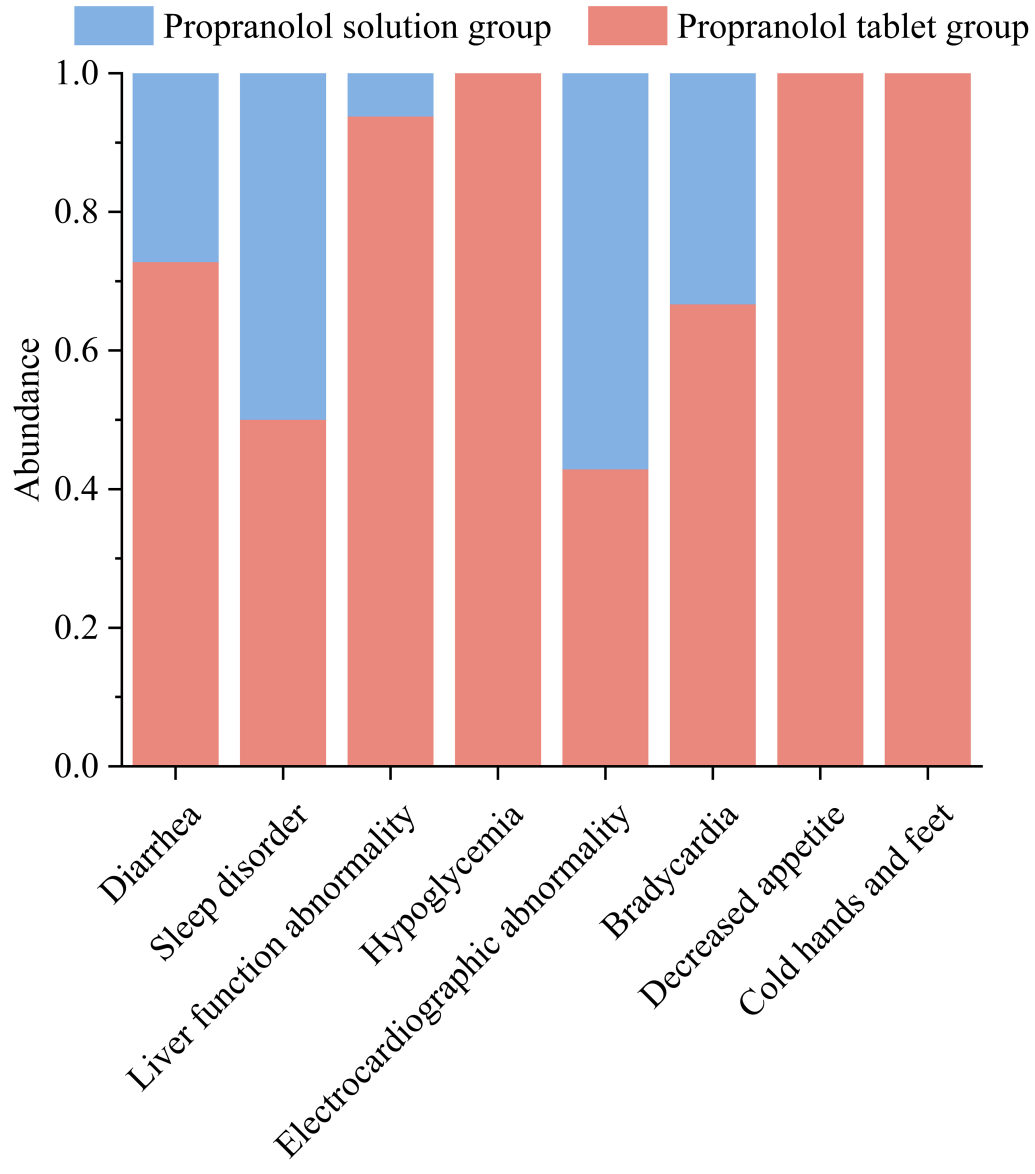
The stacked bar chart on adverse events for two groups.

The most common adverse reactions in the oral solution group were electrocardiographic abnormalities, including a prolonged QT interval with low and flat T waves in some leads in 1 patient, ST-segment elevation in some leads in 2 patients, and Left ventricular hypertrophy observed in 1 patient. The probability of liver function abnormalities, diarrhea, hypoglycemia, decreased appetite, and cold hands and feet were higher in the tablet group than in the oral solution group. All of the affected 45 patients improved after symptomatic treatment and adjustment of drug dosage without affecting the course of treatment. All data are shown in Table 3 and visualized in Figures 2, 3.

**Table 3:** Incidence of adverse events between the tablet and oral solution groups.

	*n* (%)	Diarrhea	Sleep disorder	Liver function abnormality	Hypoglycemia	Electrocardiographic abnormality	Bradycardia	Decreased appetite	Cold hands and feet
Propranolol tablet group *n* = 168	34 (20.24)	8 (4.76)	2 (1.19)	15 (8.93)	2 (1.19)	3 (1.79)	2 (1.19)	1 (0.60)	1 (0.60)
Propranolol solution group *n* = 66	11 (16.67)	3 (4.55)	2 (3.03)	1 (1.52)	0	4 (6.06)	1 (1.52)	0	0
*X* ^2^	0.39	0.01	0.96	4.09	0.79	2.98	0.04	0.40	0.40
*P*	0.53	0.94	0.33	<0.05	0.37	0.08	0.84	0.53	0.53
*OR*	0.788	0.962	2.594	0.157	0.988	3.548	1.277	0.994	0.994
*95% CI*	0.373, 1.667	0.245, 3.705	0.358, 18.806	0.020, 1.213	0.972, 1.005	0.772, 16.308	0.114, 14.325	0.982, 1.006	0.982, 1.006

## Discussion

Based on currently available data, propranolol appears to be more successful for the treatment of IH than any other option (13–15). Propranolol is a lipophilic, non-selective β-blocker, which is widely used in clinical practice to treat tachyarrhythmias, hypertension, and migraines. The mechanism of action of this non-selective β-blocker has been thoroughly investigated concerning the effects on vessels and the heart but the precise effect on IH is a matter of speculation. Currently, some scholars argue that treatment of IH with propranolol exists in three stages. During the early stage propranolol inhibits the cAMP PKA NO signal pathway, which reduces the emission of NO to slow blood flow, causes vasoconstriction, and inhibits growth of the hemangioma (16). During the middle stage, propranolol reduces endothelial cell growth and inhibits angiogenesis via VEGF and HIF-1, which in turn inhibits hemangioma proliferation (17). During the late stage, propranolol exerts a β^2^ receptor-blocking effect to inhibit hemangioma growth by inducing apoptosis of endothelial cells, perivascular cells, and other cells associated with hemangiomas (18). Based on currently available data (19), propranolol appears to be safe in patients with cardiovascular disease. The most frequently reported propranolol adverse effects include bradycardia, hypotension, and hypoglycemia, but the general safety assessment for propranolol is high (10, 20). Propranolol, which has been widely used in the past, refers to propranolol hydrochloride tablets, the treatment of which for IHs is irrational: (1) Infant anatomic functions are immature and children <7 years of age are often not able to swallow solid medications, so there are limitations to administering tablets to infants. (2) IHs are characterized by an early age at onset, a long treatment interval, and a high requirement for the precision of administration due to the dosage adjustment requirements according to infant weight. Tablets are administered by crushing and dissolving in drinking water to make a suspension, which is placed in a syringe for on-demand administration. The solubility of each propranolol tablet cannot be homogenized, making it more difficult to meet the precision of administration. (3) Infant physiologic function is immature and the safety requirements of drugs are higher than adults. Syringes with dissolved tablets are stored in refrigerators, where there are no aseptic conditions and the safety of the storage environment cannot be guaranteed.

As early as 2014, Casiraghi et al. (21) questioned the safety and efficacy of propranolol tablets in the pediatric population and concluded that the availability of propranolol hydrochloride oral solution reduced the risk of incorrect administration. In 2021, Brazil (22) conducted research on compounded cardiovascular medicines prescribed in neonatology, 75.63% of medications in the pediatric ICU need to change dosage form, through the transformation of capsules or tablets into liquid formulations, including propranolol, which represented a risk due to the presence of different excipients in the composition of these pharmaceutical products, and the lack of information on bioavailability and physical, chemical, and microbiological stability. The result shows the need for the development of medications suitable for the neonatal and children population in Brazil, which is also urgent in China.

Both domestic and international studies have focused on comparing propranolol with placebo (4) or other β-blockers, such as nadolol (8) and atenolol (23), on the safety and efficacy of IHs, while overlooking potential differences in the bioavailability between the two formulations (propranolol hydrochloride tablets and propranolol hydrochloride oral solution). Therefore, we conducted a retrospective study, collecting clinical data from 168 to 66 patients who were treated with propranolol hydrochloride tablets and oral solution throughout the period from August 2018 to February 2023, respectively, at the Children's Hospital of Soochow University to analyze efficacy and safety.

With respect to efficacy, 7.74% (13 of 168) and 6.06% of patients (4 of 66) treated with propranolol tablets and oral solution were unsuccessful with overall improvement <25%, both oral propranolol hydrochloride tablets and oral solution had significant efficacy in IH. Second, score improvement, calculated by the HASI activity score, shows that patients treated with the oral solution (69.15%) generally have a better efficacy compared with tablets (66.52%), while further analysis using a Chi-square test suggested that the difference in efficacy between the oral solution and the tablet group lacked statistical significance (*P* = 0.45). Interestingly, there were significant differences (*X*^2^ = 8.557, *P* = 0.003) in overall improvement, with mean score improvement, between 75% and 100%. 42.42% of the oral solution group achieved this, compared to 23.21% of the tablet group, which showed a better response to efficacy to some extent. While we further analyzed the duration of treatment to achieve a 75% score improvement between the two groups, there was lacking statistical significance (*P* = 0.67, HR = 1.15, 95% CI: 0.7–1.88), which indicated that a better response to efficacy in the propranolol solution group was not related to a longer duration treatment. We speculate that it may be related to differences in pharmacokinetics and absorption efficiencies. On the one hand, solution, dispersed in the medium in a particulate or molecular state, are absorbed more quickly and can exert their effects rapidly, while tablets need to be disintegrated, dispersed, dissolved, and other processes before they can be absorbed, and the efficacy is relatively slow. On the other hand, the bioavailability of solution is usually higher than that of tablets. For example, studies show that the pharmacokinetic endpoints of Trametinib solution, such as Tmax, and Cmax derived from standard non-compartmental methods at equal doses, were earlier and higher than Trametinib tablets (24). Ancient research in 1982 about the bioequivalence of two different propranolol hydrochloride tablets and a propranolol hydrochloride solution demonstrated that the three different preparations of propranolol were bioequivalent, while the plasma propranolol concentration at 0.5 h was significantly higher for the solution than for the two tablet formulations (25). In 1989, Eldon (26) used 1 × 80 mg propranolol tablet, 1 × 80 mg propranolol tablet, manufactured by 2 companies, and 80 mg of propranolol HCl in solution to conduct a pharmacokinetic study, which found that Mean AUC, Cmax, and Cmin values for solution were statistically higher than tablets, and Tmax were faster than tablets. However, the limitation of this comparison was that the 80 mg of propranolol HCI solution was dissolved in 40 mg propranolol tablets with 30 ml water, rather than the Hemangeol solution. We need up-to-date and more precise research on comparisons of propranolol tablets and off-the-shelf oral solution and the reason for this better response has to be elucidated.

In terms of safety, it was generally recognized that there are no serious systemic adverse reactions of propranolol in infantile hemangiomas, and some studies have shown that propranolol is highly safe in neonates with corrected age <5 weeks (27). In addition, there were case reports of an overdose of propranolol in infantile hemangiomas with no associated adverse effects (28). The instruction of propranolol hydrochloride solution stated that the common adverse reactions were insomnia, bronchiolitis, cold hands and feet, irritability, and diarrhea, of which the incidence of insomnia at the dose of 1.2 mg/kg/day was 17.5% and decreased appetite was 2.5%, which were higher than our study. In our study, we found that the incidence of adverse reactions was 20.24% in the tablet group and 16.67% in the solution group, but the incidence of diarrhea, liver function abnormality, hypoglycemia, decreased appetite, and cold hands and feet were higher in the tablet group than in the solution group, whereas the incidence of sleep disorders, electrocardiographic abnormality, and bradycardia were lower than in the solution group. It was statistically significant that liver function abnormalities occurred in the tablets group compared to the solution group(*X*^2^ = 4.09, *P* = 0.045). The most common causes of transient liver function abnormalities in infants and children are cytomegalovirus (CMV), and Epstein-Barr virus (EBV) infection (29). Although there was no direct evidence indicating it was drug-induced liver injury (DILI),we can't overlook the safety of home-made propranolol treatment, in which parents cracked and dissolved the tablets by themselves and placed them in syringes, and then placed them in the refrigerator for storage after on-demand feeding, which could lead to contamination to some extent. Although there had many cases of hypoglycemia (30) and even deep coma (31) caused by oral propranolol solution in patients reported in foreign countries, our data showed that only two cases of hypoglycemia were recorded in the propranolol tablet group, which were all improved after eating, and the subsequent dose of the propranolol was not adjusted. In addition, we found that 3.03% of patients with propranolol solution treatment have sleep disorders. In comparison, a study from Italy (32) found a high prevalence of sleep disorders (59%) in infants and toddlers treated with propranolol for IHs by using a specialized computerised questionnaire. There exists a significant difference, which prompts us more detailed counseling on sleep hygiene in further study.

This was a retrospective cohort study, although we had strict inclusion and exclusion criteria for propranolol therapy, which was inherently prone to selection and recall bias to a certain extent. A prospective or randomized study needs to strengthen this conclusion in future. Moreover, propranolol oral solution was not available in China until 2021, while it has been used internationally for many years, which would make this manuscript less generalizable due to differences in formulations and healthcare practices.

In sum, both propranolol hydrochloride tablets and solution had positive efficacy in high-risk infantile hemangiomas, but more patients in the solution group tended to achieve more than 75% score improvement compared to the tablet group. No life-threatening adverse reactions occurred in either group, but liver function abnormality is more likely to occur in patients treated with tablets. The incidence of adverse reactions was 16.67% in both groups, but the incidence of diarrhea, liver function abnormalities, and hypoglycemia was higher in the tablet group than in the oral solution group, while the incidence of sleep disorders and abnormal electrocardiogram results was lower than in the oral solution group.

## Conclusion

Propranolol is nowadays an effective and safe treatment for infantile hemangiomas. As pointed out in our study, propranolol hydrochloride oral solution showed better treatment efficacy and liver safety compared to propranolol hydrochloride tablets.

## Data Availability

The original contributions presented in the study are included in the article/Supplementary Material, further inquiries can be directed to the corresponding author.
